# A Longitudinal Study in Worrisome Sexual Behavior Following Sexual Abuse in Infancy or Early Childhood: The Amsterdam Sexual Abuse Case

**DOI:** 10.1007/s40653-023-00539-9

**Published:** 2023-03-31

**Authors:** Vionna M. W. Tsang, Eva Verlinden, Sonja N. Brilleslijper-Kater, Esther M. van Duin, Jos W. R. Twisk, Arnoud P. Verhoeff, Ramón J. L. Lindauer

**Affiliations:** 1 Amsterdam University Medical Centers, Department of Child and Adolescent Psychiatry, University of Amsterdam, Amsterdam, the Netherlands; 2 Amsterdam UMC, Department of Social Pediatrics, Child Abuse and Neglect Team, University of Amsterdam, Amsterdam, the Netherlands; 3 Department of Epidemiology, Health Promotion & Healthcare Innovation, Public Health Service of Amsterdam (GGD Amsterdam), Amsterdam, the Netherlands; 4 Amsterdam UMC, Department of Clinical Epidemiology and Biostatistics, VU University, Amsterdam, the Netherlands; 5 Levvel, Academic Centre for Child and Adolescent Psychiatry, Amsterdam, The Netherlands; 6 Department of Sociology, University of Amsterdam, Amsterdam, the Netherlands

**Keywords:** Worrisome sexual behavior, Sexual abuse, Very young children, Longitudinal

## Abstract

Worrisome sexual behavior (WSB) is often described as an outcome specific to child sexual abuse (CSA). Therefore, it is highly relevant to study WSB in relation to sexual abuse, especially in very young children, as it is hard to recognize sexual abuse in children who have limited verbal capacities of disclosing. Over time, literature describing WSB following CSA has gradually broadened. However, a gap remains regarding the long-term development of WSB in children who were sexually abused during infancy or very early childhood. To our knowledge, our study is the first to examine developmentally-related sexual behavior versus sexual abuse-specific behavior longitudinally in children who were sexually abused at a very young age. In total, we examined the sexual behavior, as reported by parents of 45 children who experienced early-age sexual abuse for a period of more than five years. Overall, we found that WSB is likely to be a CSA-specific and potentially long-term outcome for children who were sexually abused at a very young age. Despite the decrease in sexual abuse-specific behavior over time, the level of this behavior was still significantly high 8 years after the sexual abuse. This finding supports long-term monitoring and assessment and intervention for WSB over time. Despite these findings, it is important to note that WSB does not serve as proof of sexual abuse in children; likewise, when a child does not present with WSB, it does not indicate the absence of a substantiated history of sexual abuse.

The existence of child sexual abuse (CSA) is undeniable and its possible consequences are evident (Hornor, 2010; Putnam, 2003). Contrary to negative outcomes of CSA, such as behavioral and attachment problems, that are also generally found in other clinical samples, worrisome sexual behavior (WSB) is a consequence that has been identified as specific to CSA (Friedrich et al., 2001; Olafson, 2011; Putnam, 2003; Szanto, Lyons, & Kisiel, 2012).

Unlike most psychological outcomes following CSA, WSB is not a diagnosis nor syndrome, but regards sexual behaviors both solitary and involving another person (Kellogg, 2010) that are not accepted within societal norms (Chaffin et al., 2008). It is important to mention that sexual behavior in children in and of itself is not always worrisome (de Graaf & Rademakers, 2011). In fact, exhibiting age-appropriate sexual behavior is a healthy and crucial process in the normal development of a child (Brilleslijper-Kater & Russel, 2013), and presents in 42–73% of children before the age of 13 (Kellogg, 2010). As an example, a child looking at the genitals of others out of biological curiosity is considered typical behavior. Children learn by exploring. The development of sexual behavior in children is part of their exploration of the world. This overt explorative behavior reaches a peak between the ages of 3 and 6 years old (Brilleslijper-Kater & Russel, 2013) and is followed by a gradual decrease as children become more aware of cultural norms, which leads to adjustment in their sexual behavior (Bancroft, 2003). Whether sexual behaviors are considered normative or worrisome highly depends on the age and developmental stage (Friedrich et al., 1998; Szanto et al., 2012). Certain sexual behavior can be considered worrisome in a young child, while the same sexual behavior is not considered alarming in an older child (Vrolijk-Bosschaart et al., 2018). Normative sexual behavior is defined as that which is developmentally appropriate and does not involve coercion or distress (Jonkman et al., 2019; Kellogg, 2010). Worrisome sexual behavior is defined as sexual behavior or intrusive sexual acts that are developmentally inappropriate, involve coercion or distress, or could be harmful to the individual or others (Chaffin et al., 2008; Kellogg, 2010). It involves sexual behavior that is not typical in frequency, duration, or type of behavior, and which has a negative impact on the child or others (Silovsky et al., 2013).

WSB can provide insight into the sexual abuse history of the child and is thus important to explore and assess (Brilleslijper-Kater et al., 2004), especially when it comes to the assessment of children who may have been abused at a preverbal age, evaluating WSB is of importance (Vrolijk-Bosschaart et al., 2018). A younger age of onset is found to be predictive of worrisome sexual behavior in the aftermath of CSA (Friedrich et al., 1998, 2001; Letourneau, Schoenwald, & Sheidow, 2004; Sandnabba, Santtila, Wannäs, & Krook, 2003; Silovsky & Niec, 2002). In one study, a younger age of onset of sexual abuse (0–6 years) was found to be the most significant predictor of WSB (McClellan et al., 1996). In fact, Kendall-Tackett, Williams, and Finkelhor (1993) found the highest prevalence of WSB (35%) in the youngest children (3–5 years) in their review which compared sexually abused to non-abused children. Despite the correlation between CSA and WSB, however, there remain gaps in the literature regarding the long-term course of WSB in children sexually abused during infancy or very early childhood.

The Four Traumagenic Dynamics Model (Finkelhor, 1987), which describes a process of traumatic sexualization, betrayal, stigmatization, and powerlessness, is one of the most established and coherent conceptual frameworks in explaining the impact of CSA (Cantón-Cortés et al., 2012; Collin-Vézina, Daigneault, & Hébert, 2013). This model postulates that the conjunction of these four dynamics, which arise from sexual abuse specifically, is what makes the psychological injury from CSA unique (Finkelhor & Browne, 1985). In particular, the first of the four trauma-causing dynamics a child may experience is *traumatic sexualization*, which speaks to “when a child’s sexuality (including both sexual feelings and attitudes) is shaped in a developmentally inappropriate and interpersonally dysfunctional fashion as a result of sexual abuse” (Finkelhor & Browne, 1985, p. 531). This may lead to negative sequelae, such as impaired social abilities in modulating and expressing sexual behaviors and confusion about sexual norms and standards (Finkelhor & Browne, 1985; McClellan et al., 1996). Despite these earlier findings, and even though the research on WSB following CSA has gradually broadened, there is still limited information regarding the sexual development of those who have experienced early-age CSA (Bancroft, 2003).

One of the few studies available in the literature examined the longitudinal course of WSB in young children (n = 354) ages 3 to 8 over five years, however, it included a normative sample and a small sub-clinical sample in which the prevalence of evident sexual abuse was only 1% (n = 4) (Lussier et al., 2018). Additionally, in a previous study we conducted, we demonstrated WSB as a salient outcome following sexual abuse at a very young age, finding that around a third of the sample of children who were sexually abused at a very young age (between 0 and 3 years old) developed WSB (Vrolijk-Bosschaart et al., 2018). Both studies examined WSB at one time point after disclosure. The question that follows is whether and how this outcome changes over time, in order to provide adjusted care and support for children and their parents.

In the current study, we examined the sexual behavior of infants and toddlers with documented histories of sexual abuse longitudinally until 8 years after the last event of CSA. This longitudinal study is part of the Amsterdam Sexual Abuse Case (ASAC) study (Lindauer et al., 2014), in which a male daycare employee was suspected of sexually abusing over 150 infants and toddlers. This case is unique due to the exceptionally young age of onset of sexual abuse (0–3 years old), juridical proof with detailed documentation of the abuse, one convicted perpetrator, and no other reported forms of child abuse (Lindauer et al., 2014).

**Research question**.

The following research question will be examined: How does sexual behavior develop over a 5-year period in children who have been sexually abused at a very young age?

## Method

### Participants

The course of sexual behavior for the participants was assessed annually, from 3 to 8 years after the disclosure of the CSA, as part of the larger ongoing ASAC study (Lindauer et al., 2014). The ASAC study design and protocol have been described in detail and are available on https://bmcpsychiatry.biomedcentral.com/articles/10.1186/s12888-014-0295-7.

The participants were drawn from the group of parents from the ASAC, in which a daycare employee who also worked as a babysitter was suspected of sexually abusing 150 children in early childhood. The suspect eventually confessed to the sexual abuse of 87 very young children. In total, we examined the sexual behavior of 45 children, as reported by their parents (n = 42), over a period of five years, starting at three years after disclosure (2013–2017). The age of onset of sexual abuse was 0 to 3 years old. Table 1 presents the demographics of the sample and characteristics of the CSA. For the full overview of the participation rate, we refer to our previous paper (Van Duin et al. 2018).

**Table 1:** Demographics and characteristics of the abuse

	Mean (min - max)		SD	
Age children at abuse onset (y)	1.4 (0–3)		0.9	
Age children at first assessment (y)	6.2 (3–9)		1.3	
	* N*		*%*	
Gender child (male)	30		66.7	
*Ethnicity child*				
Native Dutch	26		57.5	
Non-native Western	12		26.7	
Non-native non Western	7		15.6	
*CSA type (n confirmed victims = 37)*				
Exposure of genitals to child	31		83.8	
Ejaculation onto child	25		67.6	
Fondling^a^	34		91.9	
Oral copulation	21		56.8	
Penetration of anus or vagina with finger, penis or sex toy	13		35.1	
*Frequency*				
Once or twice	16		43.2	
Three to ten times	15		40.5	
More than ten times	4		10.8	
Unknown	2		5.4	
Cases with pornographic evidence	15		40.5	
*Location of abuse*				
Daycare	21		56.8	
Home	12		32.4	
Both	4		10.8	

*Source Tsang et al., (2020)*

*Note*: Abuse characteristics were obtained from police reports. First assessment is 3 years after disclosure of the abuse.

^a^ Described by perpetrator as touching genitalia or masturbating the child.

### Procedure

Parents who had been in contact with either the Amsterdam University Medical Center or the Public Health Service of Amsterdam as part of aftercare following disclosure were asked for participation. The following inclusion criteria were used: (1) parents and child(ren) who had taken part in the physical and psychological examination in the Academic Medical Center, or had been in contact with the Public Health Service of Amsterdam during provision of aftercare following the disclosure; and (2) CSA was confirmed or highly suspected.

CSA was considered confirmed in cases where the perpetrator confessed and/or pornographic material depicting the child was found; CSA was considered highly suspected when a child had been in direct contact with the perpetrator and parents highly suspected sexual abuse based on symptoms warranting a valid suspicion. We decided to include both confirmed (n = 37) and highly suspected victims (n = 8). The first reason was that sexual abuse in the latter group could not be ruled out in these children. Secondly, we considered it unethical to deny participation as our study also aimed to monitor the well-being of the victims and their parents long-term (Tsang et al., 2020). Additionally, the first follow-up study showed no differences in psychopathology between the confirmed and highly suspected victims (Van Duin et al., 2018).

Written consent was obtained from parents who decided to participate in the study; separate written consent was also obtained to gather information about the psychological help parents and/or children received and to access medical and police files. As part of the study, parents agreed to provide information about their child’s sexual behavior annually for five years through a secure online questionnaire system. During the five time points, a total of seven parents withdrew consent, with five dropping out of the study after the first time point and two parents after time point four. The reasons for dropout were because the participation was ‘causing emotional distress’, ‘time consuming’, and ‘the child did not show any symptoms’. Except for travel allowance, no financial incentives were provided for parents.

### Measures

The Dutch version (Verlinden & Lamers-Winkelman, 2016) of the Child Sexual Behavior Inventory (CSBI; Friedrich, 1997), which screens for symptoms of sexual behavior in children from age 2 to 12 years, was used for symptom monitoring. Parents reported on sexual behavior that was seen at least once over the past six months. Recently published psychometric properties of the Dutch version of the CSBI have been found to demonstrate sufficient reliability and validity (Jonkman et al., 2019).

The questionnaire includes 38 items in 9 domains: boundary problems, exhibitionism, gender role behavior, self-stimulation, sexual anxiety, sexual interest, sexual intrusiveness, sexual knowledge, and voyeuristic behavior. The CSBI results in three scales: CSBI total, Developmentally-Related Sexual Behaviors (DRSB), and Sexual Abuse-Specific Items (SASI). The CSBI total scale indicates the overall level of sexual behavior and sexual behavior problems. The DRSB scale includes items indicating appropriate sexual behavior based on age and gender, such as ‘tries to look at people when they are nude’, whereas the SASI scale includes items indicating sexual abuse-related behavior, such as ‘asks others to engage in sexual acts with him or her’. Many items depend on age and gender in order to determine whether the behavior is worrisome. For instance, the item ‘stands too close’ is considered developmentally-related sexual behavior for boys until the age of 9. Elevated scores on the DRSB scale are generally related to the extent to which sexual education is provided within families and the way parents handle the subject of sexuality in the home. Elevated scores on the SASI scale may imply a history of sexual abuse. Parents received an individual report of the outcomes of the questionnaires. When these outcomes were worrisome or at clinical level, psychological support was advised. If desired, parents could request individual consultation with a clinical psychologist for specific developmental or sexual abuse-related questions.

Parents indicated the scores on a 4 point Likert scale, from 0 (never) to 3 (at least once a week). The raw scores range from 0 to 114, with a higher score indicating elevated problematic sexual behavior. The standardized T-scores, as compared to the norm group specified by gender and age, were divided into three categories: normal (T-scores below 60), subclinical (T-scores 60–64), and clinical (T-scores of 65 and higher).

#### Psychological Support

In this paper, we use the term “psychological support” for both brief, supportive contacts and trauma treatment interventions. The psychological support parents and children received varied from one or two appointments of situational support to years of full trauma treatment, and was carried out by diverse mental health care centers. Therefore, it was relevant to categorize this information by intensity of services in order to gain more insight. Categorizing was done in the following steps:

1. The first author summarized the files of children and parents, each by time point.
2. Four mental health professionals (masters level) who were not involved in this study: Nathalie Schlattmann (clinical psychologist), Irma Hein (child psychiatrist), Karen van Zon (healthcare psychologist), and Eva Bolle (healthcare psychologist) were asked to categorize the level of psychological support received in each case in the secure online system. Children and parents were rated separately for their own individual level of psychological support received. The categories included a minimal, moderate, or intensive level of psychological support. Categories were assigned based on the combination of clinical judgement, frequency of treatment, and level of care (e.g. preventive care, primary care, specialized care).
3. Consensus was defined as a unanimous score, or when at least 3 out of 4 professionals agreed. The cases scored by the professionals that did not reach consensus (psychological support child: 21%, psychological support parents: 15%) were categorized by the principal investigator (RJLL), an expert in childhood trauma, based on his own clinical experience and the reasoning of the mental health professionals.

### Ethical Considerations

The ASAC-study was approved by the Medical Ethics Review Committee of the Amsterdam University Medical Centers, Academic Medical Center.

### Data Analysis

The questionnaire data and the descriptive statistics were analyzed using IBM SPSS Statistics, version 25 (2017). The longitudinal analyses were performed with linear mixed model analyses (for continuous scores of sexual behavior) and logistic Generalized Estimating Equation (GEE) analyses (for dichotomous scores of sexual behavior: clinical/non-clinical), as the GEE analysis is found to be more accurate in analyzing dichotomous outcome scores (Twisk et al., 2017). Both analyses were used, in order to account for the dependency of the repeated observations for each child (Twisk, 2013). Both mixed model and GEE analyses were performed with STATA, version 15 (2017). For all longitudinal analyses, the severity of the CSA, age of onset, and the level of psychological support the child received were used as covariates.

Consistent with our ongoing longitudinal study (Tsang et al., 2020), the number of years after CSA were used as the time variable instead of the ages of the children, as they varied widely at each time point. Using years after CSA was more informative and relevant than the year of measurement. The group ‘3 years after CSA’ was not included in the analyses due to the small size and possible influence on the reliability of the results; however, it was used as the reference category since this group was significantly larger than the group ‘4 years after CSA’, and thus it provided more power. Since the current study is an observational longitudinal study, it was important to consider not only statistical significance, but to also focus on the development of sexual behavior and changes over time as well.

## Results

### Descriptive Statistics

We begain with exploring the development of sexual behavior over time before taking possible confounders into account. The details of the severity of the CSA and age of onset are presented in Table 1. In total, 71.1% (n = 32) of the children received some form of psychological support at one or more time points. Table 2 presents the clinical percentages of all children over five time points (i.e., clinical and subclinical scores indicating normal or worrisome range). Overall, the percentages of children above the clinical cutoff scored highest for sexual abuse-specific behavior (the SASI scale; M = 32.7%) as compared to both developmentally-related sexual behavior (DSRB; M = 7.4%) and the CSBI total scale (M = 10.3%). Additionally, the percentages on both subscales decreased over time.

**Table 2:** Clinical percentages of sexual behavior problems over the years

	T1	T2	T3	T4	T5	M
CSBI total (%)	16.3	15.0	12.8	5.3	2.9	10.3
DRSB scale (%)	9.3	15.0	7.7	5.3	0.0	7.4
SASI scale (%)	48.8	40.0	30.8	21.1	23.5	32.7

*Note*: Values represent clinical percentages on the CSBI questionnaire. Clinical scores regard both subclinical and clinical scores. DRSB = Developmentally−related Sexual Behavior. SASI = Sexual abuse−specific Items

**Fig. 1:**
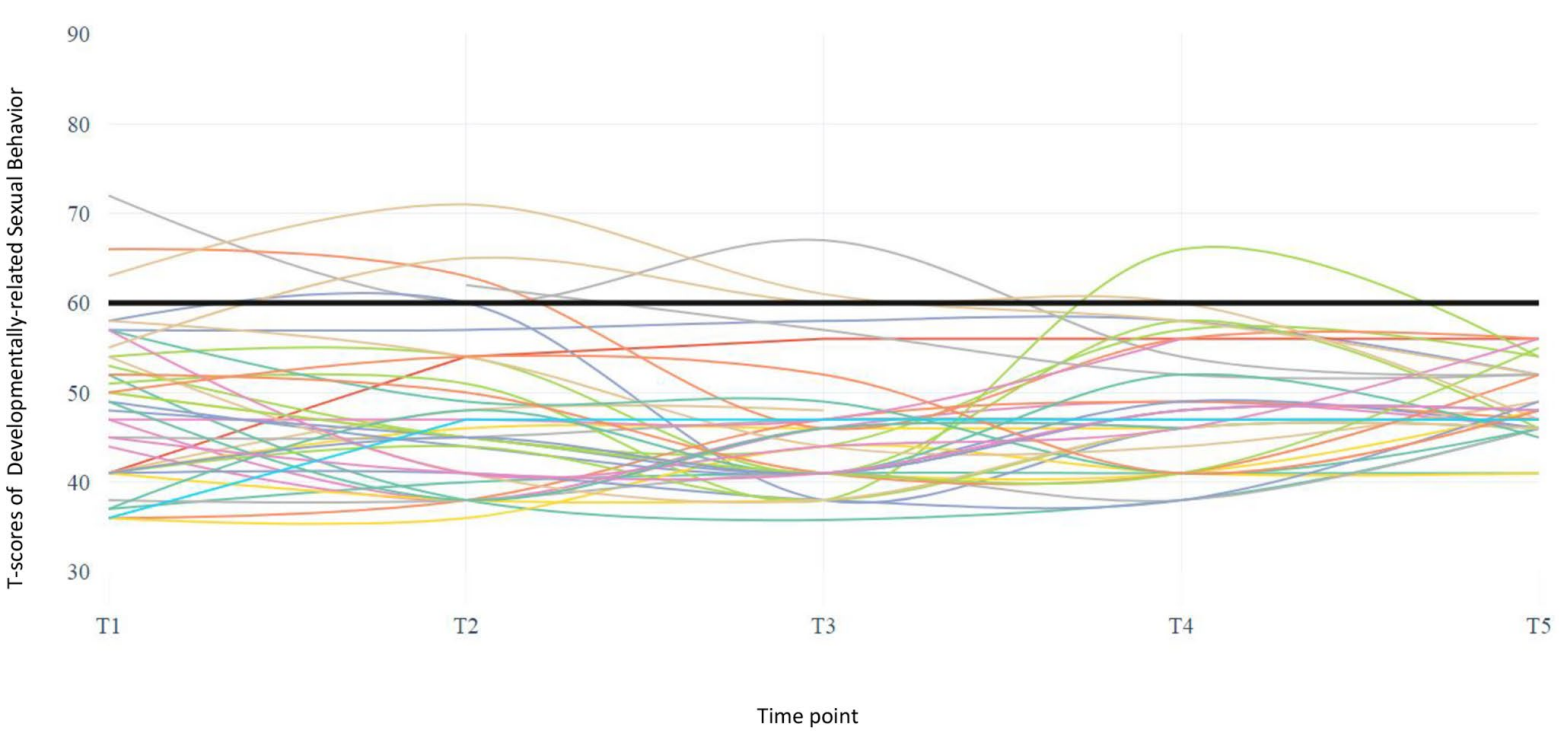
Individual trajectories of children on the Developmentally-related Sexual Behavior scale (DRSB). The black line indicates the subclinical cutoff

**Fig. 2:**
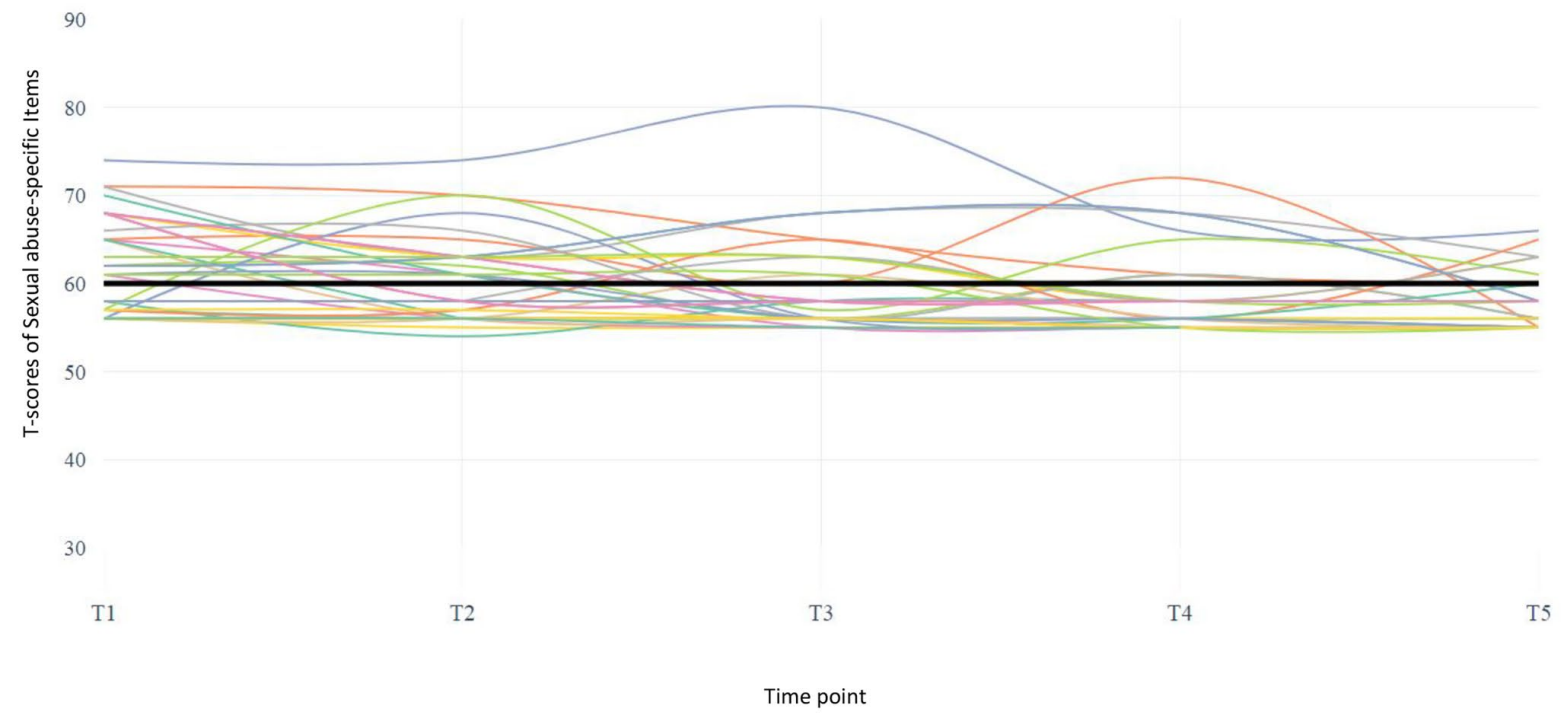
Individual trajectories of children on the Sexual abuse-specific Items scale (SASI). The black line indicates the subclinical cutoff

Figures 1 and 2 illustrate individual trajectories of the children over the five time points. Again, a minority of the children were reported to show developmentally-related sexual behavior at the subclinical and clinical level (Fig. 1), whereas a large number of children showed sexual abuse-specific behavior at the subclinical and clinical level (Fig. 2). At one or more time points, 18% of all children presented with developmentally-related sexual behavior at the clinical level, whereas 62% of all children presented with clinical sexual abuse-specific behavior at one or more time points. Even children who did not score above the subclinical cutoff showed higher T-scores on the SASI than on the DRSB.

### Longitudinal Analyses

#### Total Sexual Behavior Problems

**Fig. 3:**
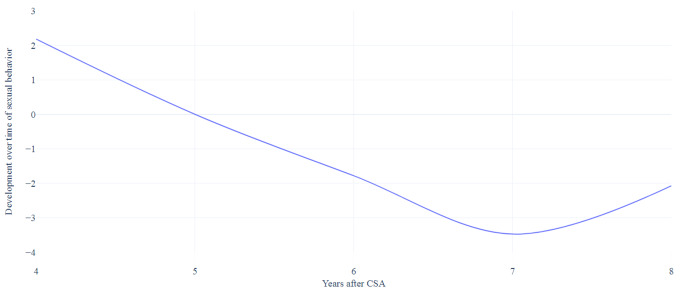
Developmental course over time of total sexual behavior problems (CSBI total scale) based on the mixed model analyses

The results of the mixed model analyses are presented in Table 3. On the CSBI total scale, the scores decreased at each time point until 7 years after CSA, with a significant decrease at that point (*b* = -3.47, 95% CI [-5.86, -1.08]). The scores increased slightly 8 years after CSA (*b* = -2.07, 95% CI [-4.68, 0.55]), as shown in Fig. 3. Additionally, GEE (see Table 4) was utilized to examine the development over time of the number of clinical cases. As the GEE analyzes only two categories, clinical cases included both those with clinical as well as subclinical scores to create the dichotomous outcome. Overall, the scores decreased. The number of clinical cases decreased significantly 7 years after CSA (*OR* = 0.13, 95% CI: [-0.03, 0.50]) and 8 years after CSA (*OR* = 0.07, 95% CI [0.01, 0.46]).

**Fig. 4:**
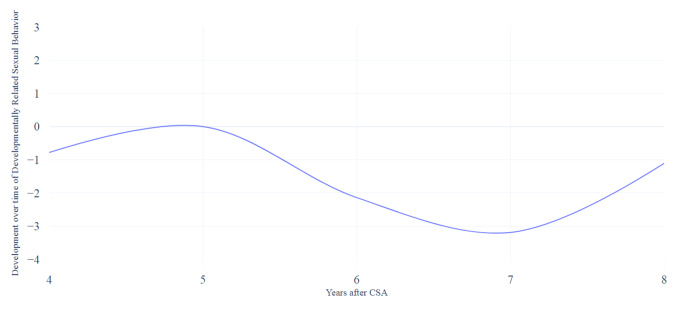
Developmental course over time of the subscale developmentally-related sexual behavior based on the mixed model analyses

**Table 3:** Results from Mixed Models of changes in sexual behavior in children

	*b (SE)*		*CI*	
*Total sexual behavior problems*				
N years after CSA (ref = 5 years)				
4	2.18 (1.41)	− 0.59		4.94
6	-1.78 (1.23)	-4.19		0.63
7	-3.47 (1.22)**	-5.86		-1.08
8	-2.07 (1.33)	-4.68		0.55
*Developmentally-related Sexual Behavior*				
N years after CSA (ref = 5 years)				
4	− 0.78 (1.56)	-3.84		2.27
6	-2.14 (1.36)	-4.81		0.54
7	-3.19 (1.35)*	-5.84		− 0.55
8	-1.11 (1.47)	-4.00		1.78
*Sexual abuse-specific Items*				
N years after CSA (ref = 5 years)				
4	1.23 (0.88)	− 0.48		2.95
6	-1.18 (0.77)	2.68		0.32
7	-1.91 (0.76)*	-3.40		− 0.43
8	-2.70 (0.83)**	-4.32		1.07

*Note*: * = p < .05, ** = p < .01. Adjusted for sexual abuse severity, age of onset, and psychological treatment child

#### Developmentally-related Sexual Behavior

The mixed model analyses (see Table 3) showed that the scores on the DRSB scale fluctuated and decreased significantly at 7 years after CSA (*b* = -3.19, 95% CI [-5.84, − 0.55]). The scores then increased slightly 8 years after CSA (*b =* -1.11, 95% CI [-4.00, 1.78]), however, not significantly, as presented in Fig. 4. The GEE showed some fluctuation in the scores on the DRSB scale, and seemed to decrease 7 years after CSA (*OR* = 0.10, 95% CI [0.01, 1.28]s, although not significantly (see Table 4).

#### Sexual abuse-specific Items

**Fig. 5:**
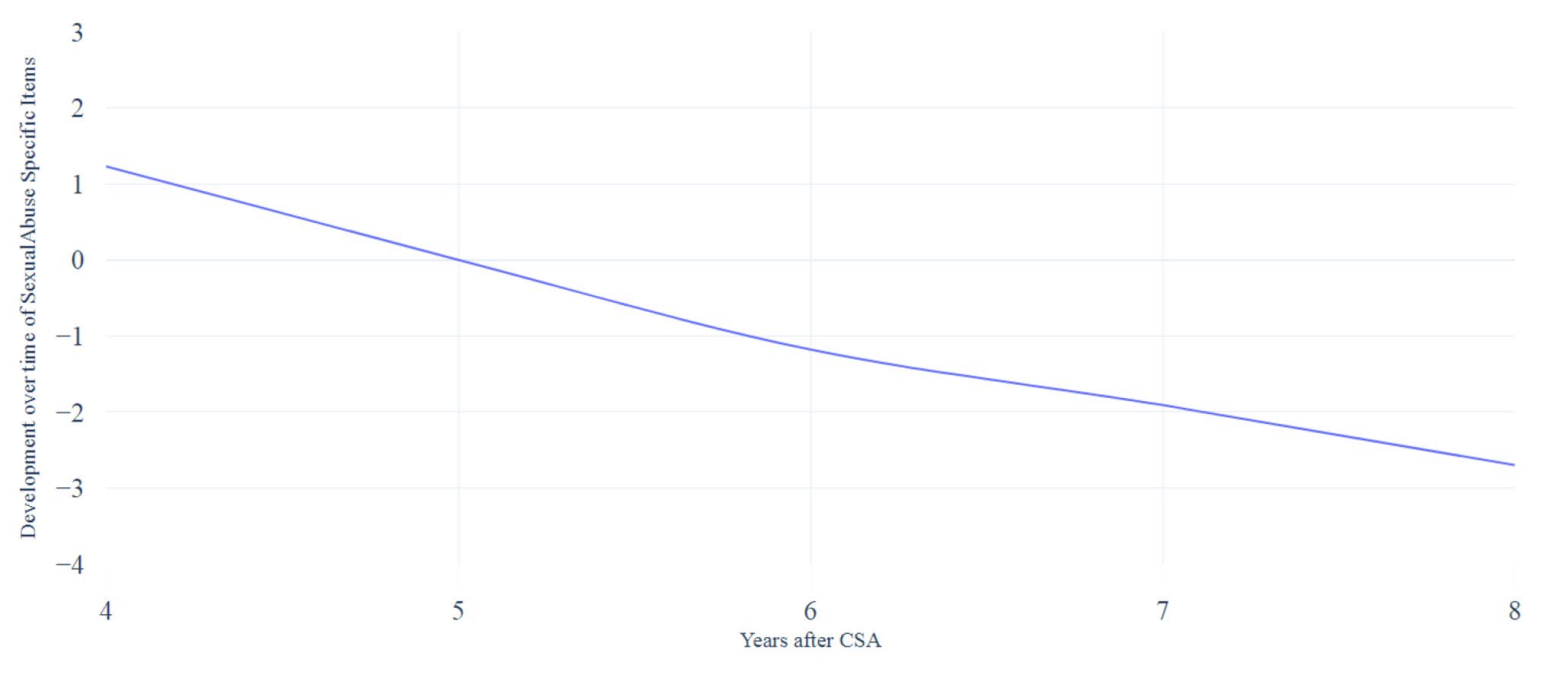
Developmental course over time of the subscale sexual abuse-specific behavior based on the mixed model analyses

The developmental course of the SASI scale, using mixed model analyses, are presented in Fig. 5. A clear decrease is visible. Scores on the SASI scale decreased significantly 7 years (*b* = -1.91, 95% CI [-3.40, − 0.43]) and 8 years (*b* = -2.70, 95% CI [-4.32, 1.07]) after CSA (see Table 3). Consistent with the mixed model analysis, the GEE (see Table 4) showed a decrease of the scores on the SASI scale, significant at 7 years (*OR* = 0.46, 95% CI [0.22, 0.95]) and 8 years (*OR* = 0.26, 95% CI [0.11, 0.63]) after CSA.

**Table 4:** Results from GEE model predicting clinical sexual behavioral problems in children

		*OR (robust SE)*			*CI*		
*Total sexual behavior problems*							
N years after CSA (ref = 5 years)							
4		0.50 (0.26)		0.18			1.39
6		0.49 (0.26)		0.17			1.41
7		0.13 (0.09)**		− 0.03			0.50
8		0.07 (0.07)**		0.01			0.46
*Developmentally-related Sexual Behavior*							
N years after CSA (ref = 5 years)							
4	0.66 (0.31)		0.27			1.65	
6	0.92 (0.37)		0.41			2.03	
7	0.10 (0.13)		0.01			1.28	
8	n/a		n/a			n/a	
*Sexual abuse-specific Items*							
N years after CSA (ref = 5 years)							
4		1.55 (0.89)		0.51			4.78
6		0.62 (0.29)		0.25			1.54
7		0.46 (0.17)*		0.22			0.95
8		0.26 (0.12)**		0.11			0.63

*Note*: * = p < .05, ** = p < .01. Adjusted for sexual abuse severity, age of onset, and psychological treatment child. n/a = not applicable: the GEE−analysis for 8 years after CSA did not lead to reliable outcomes for the DRSB scale due to the small amount of clinical cases and is therefore excluded from this table

## Discussion

This study aimed to provide insight into the developmental course of sexual behavior in children who have been sexually abused at a very young age. Our results describe the annual follow-up through eight years after CSA. To our knowledge, the current study is the first to explore sexual behavior over a long-term course in children who were sexually abused at a very young age.

Our results found that sexual abuse-specific behavior scores were higher than developmentally-related sexual behavior scores (SASI: 21–49% over five time points compared to DRSB: 0–15%). The values reported for sexual abuse-specific behavior in children at the clinical level are somewhat consistent with our earlier study (30%; Van Duin et al., 2018). A possible explanation could be as Kellogg (2010) suggests, that although the majority of sexually abused children do not develop WSB, young age of onset of sexual abuse is still a risk factor for developing WSB (Kellogg, 2010).

As to how sexual behavior changes over time, we found that developmentally-related sexual behaviors were below the clinical range and fluctuated, and they remained below the clinical range over time; sexual abuse-specific behavior scores, however, were high overall and decreased over time. Our results further support the theory that WSB is an outcome that is specific to CSA, and this continues many years after CSA. These results are not in alignment with existing literature. One of the two existing longitudinal studies (Friedrich et al., 2005) mentioned earlier looked into the continuity of WSB in preteens with no history of alleged sexual abuse and concluded that WSB did not attenuate over time. The other longitudinal study (Lussier et al., 2018) also suggests WSB can be persistent and does not necessarily attenuate. However, both study designs make it hard to compare the results as one study consisted of a sample of teenagers and a time period of one year, and the other study regards a mainly normative sample. Our study provides support to the idea that sexual abuse-specific behavior may be a specific outcome following sexual abuse in very young children. The percentages of developmentally-related sexual behavior, on the other hand, seemed to remain low and to be more comparable to the norm. In the context of our sample, with victims of extrafamilial sexual abuse with no other reported forms of child maltreatment, we found that sexual abuse-specific behavior decreased over the years. However, at the fifth time point, almost a quarter of the children continued to show sexual abuse-specific behavior at a clinical level. This result may be explained by the Traumagenic Four dimensions model (Finkelhor, 1987) which posits that worrisome sexual behaviors are a result of confusion about sexual norms and standards which stem from a traumatic sexual experience, and may have lasting impacts on the sexual development of the child.

### Strengths and Limitations

An important strength of this study is that, to our knowledge, this is the first longitudinal study focusing on the development of sexual behavior in children who were sexually abused at a very young age. According to McClellan et al. (1996), many studies targeting sexual abuse are retrospective in design and include samples that are heterogeneous in factors such as severity of the abuse, age of onset, the victim relationship to the offender, and other forms of child maltreatment, making it a challenge to find samples to study individual abuse characteristics at an independent level. The uniqueness of the ASAC-study is due to the homogeneous group of children analyzed: all were very young (0–3 years of age) at the onset of the sexual abuse, and all cases concern extrafamilial CSA by one convicted perpetrator. Additionally, the CSA took place under the same circumstances (at the daycare center or at home), detailed documentation of the abuse was available, and no other form of child maltreatment was reported. Furthermore, we have used the CSBI, which is the most widely used instrument to assess sexual behavior in children and is one of the few available instruments found to be reliable and valid in national and international studies for assessing WSB in children.

There are also some limitations. The first and most important one is the relatively small sample size. We took this into account by carefully choosing the statistical methods and controlling for missing data in the longitudinal study design (Twisk, 2013). With these steps, we have tried to maximize the reliability of our study. A second limitation is the lack of a control group. One mitigating factor to that, however, is that the CSBI has been contrasted against norm groups, nationally as well as internationally, and is corrected for age and gender. Additionally, another limitation to consider is that we have assessed WSB using a questionnaire administered to parents and not through an interview or observation. However, reliable and valid interviews or observation methods for sexual behavior problems are limited. Besides, parental reports have been previously identified as the most common and generally used method to gain insight into the sexual behavior of children (Friedrich et al., 1998). As de Graaf and Rademakers (2011) demonstrated in their review, there is no single best method to study child sexual behavior, as it is influenced by the child’s memory, language, and social development. Specific research methods can assist in gaining insight into the sexual behavior of children and can be of added value, depending on the question and ages of the children that are studied (de Graaf & Rademakers, 2011). We have tried to minimize these constraints by taking potential confounders into account, namely, sexual abuse severity, age of onset, and psychological treatment of the child. In our sample, there were no other reported forms of child maltreatment, nor continuous changes in caregivers.

Lastly, the generalizability of these results is subject to certain limitations due to the limited sample size and based on one sexual abuse case study, meaning the results should be interpreted with caution. Nevertheless, these findings contribute to our understanding of the developmental course of sexual behavior in children who have been sexually abused at a very young age, and takes a step towards filling this gap in the literature.

### Conclusion and Clinical Implications

To conclude, we can infer that WSB is likely a CSA-specific outcome that applies to children who have been sexually abused at a very young age. This also aligns with our earlier findings, which showed that WSB following CSA is a salient outcome (Van Duin et al., 2018; Vrolijk-Bosschaart et al., 2018). Our study has shown that WSB persists over time as sexual abuse-specific behavior starts decreasing significantly from 7 years after CSA, and still presents 8 years after CSA. Furthermore, the clinical percentages over the five time points were at least two times higher in sexual abuse-specific items than in developmentally-related behavior. The descriptive statistics have shown a clear difference between the outcomes of the two subscales. As developmentally-related sexual behavior fluctuates over time, sexual abuse-specific behavior seems to decrease over time. Despite the decrease, at the fifth time point, still 23% of the children showed sexual abuse-specific behavior at a clinical level. Consequently, this result indicates the need for long-term monitoring as well as evidence-based treatment after CSA.

Despite the high level of WSB, in our study, none of the psychological support sought for the children was primarily focused on decreasing WSB. This corroborates the idea of Lussier et al. (2018) that WSB is not routinely recognized and assessed by mental health professionals. A clinical implication of this study suggests that early treatment focusing on assessment of and intervention for WSB is appropriate and efficacious in cases where there are elevated WSB scores, as our study has shown it to be persistent over time. It is important to remember, however, that WSB does not serve as proof of sexual abuse in children; likewise, when a child does not present with WSB, it does not indicate the absence of a substantiated history of sexual abuse.

### Future Perspectives

Future work is required to establish the further developmental course of WSB. Our longitudinal study is ongoing and will continue for several more years. We propose that further research be undertaken in the use of multi-informant data, e.g. the inclusion of self-report by children. Also, further work needs to be carried out to establish how different types of CSA, including intrafamilial CSA, impacts the development and longevity of WSB. In conclusion, we hope that our study will serve as a base for future research on the developmental course of WSB in relation to CSA in very young children.

## References

[CR1] Bancroft, J. H. (2003). *Sexual development in childhood* (7 vol.). Indiana University Press.

[CR2] Brilleslijper-Kater, S. N., Friedrich, W. N., & Corwin, D. L. (2004). *Sexual knowledge and emotional reaction as indicators of sexual abuse in young children: Theory and research challenges*. Child Abuse & Neglect. 10.1016/j.chiabu.2004.06.005 15519432

[CR3] Brilleslijper-Kater SN, Russel IMB, van de Putte EM, Lukkassen IMA, Russel IMB, Teeuw AH (2013). Normale en zorgelijke seksuele ontwikkeling bij kinderen tot en met 12 jaar. Medisch handboek kindermishandeling.

[CR4] Cantón-Cortés D, Cortés MR, Cantón J (2012). The role of traumagenic dynamics on the psychological adjustment of survivors of child sexual abuse. European Journal of Developmental Psychology.

[CR5] Chaffin M, Berliner L, Block R, Johnson TC, Friedrich WN, Louis DG, Silovsky JF (2008). Report of the ATSA task force on children with sexual behavior problems. Child Maltreatment.

[CR6] Collin-Vézina D, Daigneault I, Hébert M (2013). Lessons learned from child sexual abuse research: Prevalence, outcomes, and preventive strategies. Child and adolescent psychiatry and mental health.

[CR7] de Graaf H, Rademakers J (2011). The psychological measurement of childhood sexual development in western societies: Methodological challenges. Journal of Sex Research.

[CR8] Finkelhor D (1987). The trauma of child sexual abuse: Two models. Journal of interpersonal violence.

[CR9] Finkelhor, D., & Browne, A. (1985). The traumatic impact of child sexual abuse: a conceptualization. *Am J Orthopsychiatry, 55*(4), 530–541. Retrieved from http://www.ncbi.nlm.nih.gov/pubmed/4073225 10.1111/j.1939-0025.1985.tb02703.x 4073225

[CR10] Friedrich WN (1997). Child sexual behavior inventory: Professional manual.

[CR11] Friedrich WN, Baker AJ, Parker R, Schneiderman M, Gries L, Archer M (2005). Youth with problematic sexualized behaviors in the child welfare system: A one-year longitudinal study. Sexual Abuse: A Journal of Research and Treatment.

[CR12] Friedrich WN, Fisher J, Broughton D, Houston M, Shafran CR (1998). Normative sexual behavior in children: A contemporary sample. Pediatrics.

[CR13] Friedrich WN, Fisher JL, Dittner CA, Acton R, Berliner L, Butler J, Wright J (2001). Child sexual behavior inventory: Normative, psychiatric, and sexual abuse comparisons. Child Maltreatment.

[CR14] Hornor G (2010). Child sexual abuse: Consequences and implications. Journal Of Pediatric Health Care : Official Publication Of National Association Of Pediatric Nurse Associates & Practitioners.

[CR15] Corp IBM (2017). Released 2017. IBM SPSS Statistics for Windows, Version 25.0. Armonk.

[CR16] Jonkman CS, Verlinden E, Punt DJ, Lamers-Winkelman F (2019). The child sexual behavior inventory: Reliability and validity in a dutch normative and clinical sample. Child Abuse & Neglect.

[CR17] Kellogg ND (2010). Sexual behaviors in children: Evaluation and management. American family physician.

[CR18] Kendall-Tackett KA, Williams LM, Finkelhor D (1993). Impact of sexual abuse on children: A review and synthesis of recent empirical studies. Psychological Bulletin.

[CR19] Letourneau EJ, Schoenwald SK, Sheidow AJ (2004). Children and adolescents with sexual behavior problems. Child Maltreatment.

[CR20] Lussier P, McCuish E, Mathesius J, Corrado R, Nadeau D (2018). Developmental trajectories of child sexual behaviors on the path of sexual behavioral problems: Evidence from a prospective longitudinal study. Sexual Abuse: A Journal Of Research And Treatment.

[CR21] McClellan J, McCurry C, Ronnei M, Adams J, Eisner A, Storck M (1996). Age of onset of sexual abuse: Relationship to sexually inappropriate behaviors. Journal Of The American Academy Of Child And Adolescent Psychiatry.

[CR22] Olafson E (2011). Child sexual abuse: Demography, impact, and interventions. Journal of Child & Adolescent Trauma.

[CR23] Putnam FW (2003). Ten-year research update review: Child sexual abuse. Journal Of The American Academy Of Child And Adolescent Psychiatry.

[CR24] Sandnabba NK, Santtila P, Wannäs M, Krook K (2003). Age and gender specific sexual behaviors in children. Child Abuse & Neglect.

[CR25] Silovsky JF, Niec L (2002). Characteristics of young children with sexual behavior problems: A pilot study. Child Maltreatment.

[CR26] Silovsky, J. F., Swisher, L. M., Widdifield, J., & Turner, V. L. (2013). *Handbook of child and adolescent sexuality: Chap. 20. Children with sexual behavior problems*. Elsevier Inc. Chapters.

[CR27] StataCorp (2017). Stata Statistical Software: Release 15. College Station.

[CR28] Szanto L, Lyons JS, Kisiel C (2012). Childhood trauma experience and the expression of problematic sexual behavior in children and adolescents in state custody. Residential Treatment for Children & Youth.

[CR29] Twisk, J. W. R. (2013). *Applied longitudinal data analysis for epidemiology: A practical guide*. Cambridge University Press.

[CR30] Twisk, J. W. R., de Vente, W., Apeldoorn, A., & de Boer, M. (2017). Should we use logistic mixed model analysis for the effect estimation in a longitudinal RCT with a dichotomous outcome variable?Epidemiology, Biostatistics and Public Health, *14*(3).

[CR31] Verlinden E, Lamers-Winkelman F (2016). CSBI: Vragenlijst over seksueel gedrag bij jonge kinderen.

[CR32] Vrolijk-Bosschaart TF, Brilleslijper-Kater SN, Verlinden E, Widdershoven GAM, Teeuw AH, Voskes Y, Lindauer RJL (2018). A descriptive mixed-methods analysis of sexual behavior and knowledge in very young children assessed for sexual abuse: The ASAC Study. Frontiers in Psychology.

